# Pharmacokinetic Characterization and Comparative Bioavailability of an Innovative Orodispersible Fixed-Dose Combination of Ivermectin and Albendazole: A Single Dose, Open Label, Sequence Randomized, Crossover Clinical Trial in Healthy Volunteers

**DOI:** 10.3389/fphar.2022.914886

**Published:** 2022-07-14

**Authors:** Jaime Algorta, Alejandro Krolewiecki, Filipe Pinto, Silvia Gold, Jose Muñoz

**Affiliations:** ^1^ Laboratorios Liconsa, Departamento Médico, Barcelona, Spain; ^2^ Instituto de Investigaciones de Enfermedades Tropicales (IIET-CONICET), Sede Regional Orán, Universidad Nacional de Salta, Orán, Argentina; ^3^ ISGlobal, Hospital Clínic-Universitat de Barcelona, Barcelona, Spain; ^4^ BlueClinical Phase 1, Hospital de Prelada, Porto, Portugal; ^5^ Fundación Mundo Sano, Buenos Aires, Argentina; ^6^ Barcelona Institute for Global Health (ISGlobal), Hospital Clinic-University of Barcelona, Barcelona, Spain

**Keywords:** pharmacokinetics, bioavability, ivermectin, albendazole, helminitiasis

## Abstract

Soil-transmitted helminths are intestinal worm diseases transmitted through the soil. Available treatments are albendazole and/or ivermectin. The co-administration of existing drugs is an appropriate strategy. A fixed-dose combination adds practical advantages mainly considering mass drug administration. The aim is to characterize pharmacokinetics and to evaluate the comparative bioavailability of an innovative fixed-dose combination of ivermectin/albendazole 18/400 mg compared with the marketed references. Seventy-eight healthy volunteers were included in this laboratory-blinded, randomized, three-treatment, three-period crossover study. Each subject received a single dose of ivermectin/albendazole 18/400 mg (1 tablet); ivermectin 3 mg (6 tablets); and albendazole 400 mg (1 tablet). Serial blood samples for the pharmacokinetic analysis were obtained pre-dose and up to 72 h post-dose. Plasma concentrations of ivermectin H2B1a, ivermectin H2B1b, albendazole, and albendazole sulfoxide were analyzed by LC-MS/MS. Pharmacokinetic parameters were estimated by a non-compartmental analysis and bioavailability compared through a bioequivalence analysis. Safety and tolerability were assessed throughout the study. Main pharmacokinetic parameters of the fixed combination were estimated for both, ivermectin [C_max_ (mean, confidence interval): 86.40 (30.42–39.23) ng/ml; AUC_0-72_ (mean, CI): 1,040 (530–1,678) ng·h/mL; t_max_ (median, min., and max.); 4.50 (2.50–5.50)] and albendazole [C_max_ (mean, CI): 22.27 (1.89–111.78) ng/ml; AUC_0-72_ (mean, CI): 94.65 (11.65–507.78) ng·h/mL; t_max_ (median, min., and max.): 2.50 (1.00–12.00) h]. The 90% confidence interval of the geometric mean ratios demonstrated the bioequivalence in the case of ivermectin (C_max_: 110.68%–120.49%; AUC_0-72_: 110.46%–119.60%) but not in the case of albendazole (C_max_: 53.10%–70.34%; AUC_0-72_: 61.13%–76.54%). The pharmacokinetic profile of a new fixed-dose combination of ivermectin and albendazole was characterized. The bioequivalence versus the reference ivermectin was demonstrated, though bioequivalence versus albendazole was not shown. The three medications analyzed were well tolerated. The results allow the advancement to the next phase of the clinical program to demonstrate efficacy and safety in patients affected by soil-transmitted helminths.

**Clinical Trial Registration:** https://www.clinicaltrialsregister.eu/ctr-search/search/, identifier Nr. 2020-003438-19

## Introduction

Soil-transmitted helminths (STH) refer to a group of intestinal worm diseases transmitted through contaminated soil: *Ascaris lumbricoides*, *Strongyloides stercoralis*, *Trichuris trichiura* (also known as whipworms), and *Ancylostoma duodenale* and *Necator americanus* (or hookworms). STH disease is usually mild and proceeds without noticeable symptoms, except for heavy infections which can cause abdominal pain, diarrhea, anemia, protein loss, rectal prolapse, and others. In children, physical and cognitive growth retardations are also frequent.

More than a quarter of the world’s population is at risk of infection with STH, having the highest prevalence in regions with a warm and moist climate and in areas of the lowest socioeconomic status with poor sanitation and hygiene, which facilitates the transmission. These diseases are the most prevalent of all neglected tropical diseases worldwide and disproportionately affect impoverished populations, causing significant morbidity in pre-school and school-age children (Jourdan et al., 2017). The 2017 Global Burden of Disease report ranks STH as the disease that poses the greatest burden of years lived with disability, with an estimation of 1.66 million years (The 2017 Global Burden of Disease et al., 2018).

In addition to interventions to improve sanitation and hygiene measures, the core intervention for reducing morbidity and transmission of STH is the preventive chemotherapy as periodic mass drug administration campaigns (Gabrielli et al., 2011). This implies the large-scale distribution of anti-helminthic drugs, typically with a single dose, to populations at risk without a previous diagnosis.

Currently, available treatments against STH are benzimidazole drugs, albendazole (ALB), and mebendazole, with proven safety and efficacy. Albendazole exhibits larvicidal, ovicidal, and vermicidal activities, and exert its anti-helmintic action in the intra-intestinal region. Albendazole is poorly absorbed (<5%) though a sufficient amount is absorbed as to assess its plasma quantification and subsequent bioavailability analysis. Albendazole undergoes an extensive first-pass metabolism, and its primary metabolite is albendazole sulfoxide. The efficacy of ALB is very satisfactory against *A. lumbricoides*, showing a cure rate close to 100%, and also remains above 90% and around 80% for hookworms. However, its efficacy against other STH species is poor, estimating as low as 31% for *T.* trichiura (Moser et al., 2017). It is particularly alarming that the efficacy against *T. trichiura* seems to have decreased from 30 to 15% in the last 16 years (Moser et al., 2017), which can be attributed to the development of drug resistance. In addition, the currently recommended regimens against STH show very low efficacy against *S. stercoralis* (Krolewiecki et al., 2013), which remains largely untreated with the current World Health Organization (WHO) strategy. Given this situation, there is an increasing need to identify new therapeutic regimens with improved efficacy while maintaining or improving safety, and subsequently provide evidence that may support the revision of the current WHO strategy, if the transmission–interruption goals are to be achieved (Anderson et al., 2015; Lo et al., 2016).

Ivermectin (IVM) is a derivative of the avermectins, a family of the macrocyclic lactones. It is a mixture containing at least 90% of 5-O-demethyl-22,23-dihydroavermectin A1a and less than 10% of 5-Odemethyl-25-de (1-methylpropyl)-22,23-dihydro-25-(1-methylethyl) avermectin A1a, generally referred to as 22,23-dihydroavermectin B1a and B1b, or H2B1a and H2B1b, respectively. Once absorbed, ivermectin is metabolized in the liver. Then, ivermectin and its metabolites are excreted almost exclusively in the feces over an estimated period of 12 days, with less than 1% of the administered dose being excreted in the urine. Ivermectin is a highly effective anti-helminthic agent, used in animals and humans against several diseases, including onchocerciasis, lymphatic filariasis, strongyloidiasis, scabies, and STH. Considering this broad efficacy spectrum, ivermectin would be an attractive agent for a combined treatment approach in settings where multi-parasitism is the norm (Richards, 2017).

Both drugs, IVM and ALB, are included in the World Health Organization’s List of Essential medicines for adult and children for the treatment of STH and other indications (World Health Organization, 2022a; 2022b). The use of a co-administration therapy with existing drugs against STH has been identified as a strategy that could offer a solution to the drawbacks and risks of the current strategy of monotherapy. In particular, the co-administration of ALB and IVM has several proven advantages, such as an improved efficacy against *T. trichiura* and *S. stercoralis* (Echazú et al., 2017) or a decreased risk of drug resistance due to the two different mechanisms of action (Vercruysse et al., 2011). The use of a fixed-dose combination adds practical advantages over co-administration having simpler storage or transport, easier dispensation, and higher acceptability. Moreover, the combination therapy is of special interest in the case of the strategy of mass drug-administration campaigns, a key component of programs aimed at controlling STH in areas of high prevalence, where medication is massively administered without a previous individual diagnosis.

The Stopping Transmission Of intestinal Parasites (STOP) project is a consortium of African and European public and private partners, funded by the European and Developing Countries Clinical Trials Partnership (EDCTP), an EU-supported partnership between the governments of 14 European and 18 African countries, funding clinical research for medical tools to detect, treat, and prevent poverty-related infectious diseases in sub-Saharan Africa. The STOP project was created with the aim of developing a fixed-dose combination of ivermectin and albendazole that facilitates its administration under a mass drug administration strategy. The development includes a strength to be used in adult populations (ALB/IVM 400 mg/18 mg) and another strength for children (ALB/IVM 400 mg/9 mg). This is the first clinical trial conducted by the consortium and was designed to characterize the pharmacokinetic profile and to evaluate the comparative bioavailability of the fixed-dose combination versus each of the reference products, already marketed, as required by the appropriate European Medicines Agency (EMA) guidelines (European Medicines Agency, 2017).

## Materials and Methods

### Study Design and Ethics

The design corresponds to a human pharmacology (phase I), single-dose, open-label, laboratory-blinded, sequence-randomized, six-sequence, three-treatment, three-period crossover study conducted in healthy volunteers under light meal conditions. The clinical study was carried-out between February and March 2021, at a single investigational center (BlueClinical Phase I, Hospital da Prelada, Porto, Portugal). Prior to the initiation of the study, its design and outcomes (as well as the full clinical development program) were discussed with EMA through a scientific advice procedure.

Before the beginning of the study, the protocol and written subject information and the informed consent form were authorized by the Portuguese Ethics Committee for Clinical Research (01/Sep/2020; 2020-RP-11–15) and approved by the National Authority for Medicines and Health Products—INFARMED (21/Aug/2020; 485/VPCD/2020). The study was conducted in compliance with the approved protocol and according to Good Clinical Practices, the Declaration of Helsinki, the European Medical Agency guidelines, and the applicable Portuguese laws and regulations. The study was handled in accordance with the existing standard operating procedures and monitored by external trained monitors.

All subjects voluntarily accepted to participate and signed the informed consent prior to any study-related activity. Each prospective participant received a full explanation of the objectives, procedures, restrictions, and potential hazards of the study. Once this information was provided to the subject, the prospective participant was required to read the information form and ask any questions about its contents. Only after the physician in charge had the conviction that the subject was aware of the implications of participating in the study, the subject was requested to confirm his/her willingness to participate by signing and dating the informed consent.

### Study Population

The study subjects were healthy male and non-pregnant female volunteers, aged 18–65 years. All participants must have a normal body mass index (18–25 kg/m^2^), a normal medical history and physical examination, without any significant diseases (gastrointestinal, hepatic, renal, respiratory, cardiovascular, metabolic, skin, immunological, or hormonal). Vital signs, 12-lead electrocardiogram, and clinical laboratory tests (hematology, general biochemistry, serology, and urinalysis) were also performed during screening. Women of childbearing potential had to use one contraceptive method. Urine drug-abuse screens for amphetamines, cannabinoids, benzodiazepines, cocaine, opioids, barbiturates, and cotinine, as well as breath alcohol tests and pregnancy tests (females only) and SARS-CoV-2 testing were carried out during screening and at the admission to each hospitalization period.

### Treatments

Subjects were allocated to receive all the three investigational treatments (crossover design) in accordance with a randomized sequence (i.e., the order of receiving each treatment along the three study periods). A total of six sequences were computer-generated and 13 subjects were allocated to each of them in accordance with a sequence-balanced randomization. The treatments were: six tablets of ivermectin 3 mg (Stromectol^®^, Merck, Sharp & Dohme BV) and albendazole 400 mg tablets (Eskazole^®^, Smith Kline & French Laboratories Ltd.) as references; one table of Ivermectin/albendazole 18/400 mg, orodispersible tablets (Laboratorios Liconsa, Spain) as the experimental drug. This experimental formulation was developed by the Pharmaceutical Development Department at Laboratorios Liconsa S.A in two strengths for use at different body weights: 9/400 mg and 18/400 mg. Both formulations are alike, as a white, round, biconvex tablet of approximately 16 mm of diameter and debossed in one side with 9/400 and 18/400, respectively. The medicines were developed to be orodispersible–chewable tablets in order to be administrated with or without water and by adding a flavor to mask the bitter flavor of albendazole. The excipients selected to develop this product were chosen considering the pharmaceutical form target. All excipients used are water-soluble to enhance the orodispersible–chewable properties. One of the goals of this formulation is to have a similar dissolution profile to the monocomponent reference products. Formulation and manufacturing developments were improved accordingly to comply with the similarity calculation (f2) versus the reference products.

All the treatments were given as a single dose, under the direct supervision of a member of the investigational team, who confirmed the intake by mouth inspection. The FDC was placed in the tongue where it disintegrated before being swallowed without water, while single active products were swallowed whole with 240 ml of water. A washout time of at least 28 days between periods was established. Subjects were requested to abstain from taking any medicinal products, vitamins, food supplements, and herbal supplements (including St John’s Wort), from 14 days prior to admission to the first study period until the end-of-study. No medication other than the investigational products was allowed during the study, unless absolutely required for treatment of adverse events. In case a subject was administered another drug, its use was to be reported and its possible impact on the study outcome was to be assessed by the investigator.

In each period, subjects were housed at the clinical research facilities for each of the three study periods and remained confined from at least 11 h before dosing until after the 24-h post-dose procedures. The corresponding medication was administered 30 min after a standard light meal containing 330 kcal, 13% fat. Meals were standardized and identical in composition throughout the study periods. Subjects were requested to abstain from consuming food or beverages that could interfere with drug metabolism from 7 days prior to the admission of each study period until the last blood sample collected in each study period. Then, ambulatory samples were taken 36, 48, and 72-h after each dosing.

### Pharmacokinetic Evaluation

In each period, a total of 21 venous blood samples were obtained pre-dosing and at 1, 2, 2.5, 3, 3.3, 3.6, 4, 4.2, 4.5, 4.8, 5, 5.5, 6, 8, 10, 12, 24, 36, 48, and 72 h post drug administration for the plasma quantification of ivermectin H2B1a, ivermectin H2B1b, albendazole, and albendazole sulfoxide. The subject’s total volume of blood withdrawn during the study, including 16 ml required for safety tests, was approximately 416 ml. The total blood donation could be slightly higher if repeat blood samples were required for safety assessments.

Samples were maintained at −80°C until shipment to the bioanalytical laboratory that developed, validated, and carried out both analytical methods (Kymos Pharma Services SL, Barcelona, Spain). A bioanalysis was carried out in accordance with the applicable international guidelines (CEDER Industry and EMA guidance on the validation of bioanalytical methods). The quantifications of ivermectin B1a and ivermectin B1b in human plasma samples were carried out by LC-MS/MS after liquid–liquid with Novum SLE plates. Doramectin was used as the internal standard. The calibration range of the method was defined from 1 to 200 ng/ml for ivermectin B1a and from 0.04 to 4 ng/ml for ivermectin B1b. The validated diluted factor was 10-fold for both compounds. The 96.6 and 76.8% respectively of the incurred sample re-analysis met the acceptance criteria. The quantifications of albendazole and albendazole sulfoxide in human plasma samples were carried out by LC-MS/MS after liquid–liquid with ethyl acetate. Albendazole-d7 and albendazole sulfoxide-d7 were used as internal standards, respectively. The calibration range of the method was defined from 2 to 600 ng/ml for albendazole and from 3 to 3,000 ng/ml for albendazole sulfoxide. The validated diluted factor in both methods was 10-fold for albendazole and albendazole sulfoxide. The 99.0 and 99.5% respectively of the incurred sample re-analysis met the acceptance criteria, hence demonstrating the reliability of the reported plasma human concentrations.

For each of the four analytes, concentration/time graphs were plotted, and the main pharmacokinetic parameters were calculated: maximum observed plasma concentration (C_max_) and area under plasma concentration versus time curve from time zero to truncated at 72 h post-administration (AUC_0-72_) in the case of ivermectin H2B1a, ivermectin H2B1b, or to 48 h post-administration (AUC_0-t_) for albendazole and albendazole sulfoxide. In addition, the time of occurrence of C_max_ (t_max_), apparent terminal elimination rate constant (λz), and apparent terminal half-life (t1/2) values are also provided. The parameters were estimated using a non-compartmental approach. The trapezoidal rule was used to estimate the AUC.

Comparative bioavailability was assessed by means of a bioequivalence analysis. In brief, an analysis of variance (ANOVA) was performed on the ln-transformed primary pharmacokinetic parameters, C_max_ and AUC_0-72_ for ivermectin and AUC_0-t_ for albendazole. The data were subsequently analyzed by means of an analysis of variance (ANOVA) model. The terms included in the ANOVA model were sequence, subject nested within sequence, period and formulation, which were used as the fixed effects. Sequence, period, and formulation were assessed at the 5% two-sided level. For this purpose, the ivermectin/albendazole FDC is considered “Test” and each of the single active substance as “Reference.” The Test-to-Reference geometric mean ratio (GMR) and its corresponding 90% confidence interval (CI) were calculated for the ln-transformed primary pharmacokinetic parameters. Bioequivalence is inferred if the 90% CIs for the Test-to-Reference GMR calculated for the ln-transformed primary pharmacokinetic parameters are all within the 80.00–125.00% acceptance interval. In accordance with international guidelines, bioequivalence is based exclusively on the determination of ivermectin H2B1a (comprising more than 90% of the total ivermectin) (World Health Organization, 2021a) and albendazole (the parent compound) (World Health Organization, 2021b), and data corresponding to ivermectin H2B1b and albendazole sulfoxide are considered as supportive.

### Safety Evaluation

For safety evaluation, the occurrence of adverse events (AE) and vital signs (blood pressure and heart rate) were monitored throughout the study. Clinical laboratory tests were performed at the screening and the end of the study (after the collection of the last blood sample of the study). Follow-up of AEs still ongoing at the end-of-study or 30 days after a premature study discontinuation for a given subject were to be extended until they were no longer considered clinically relevant. All AEs were summarized by the MedDRA dictionary (version 23.1), classified by system organ class and the preferred term, and assessed by seriousness (Serious/Non Serious), maximum severity (Mild/Moderate/Severe), drug relationship (Reasonably Possible/Not Reasonably Possible), expectedness, action taken, and outcome.

### Statistical Methods and Sample Size Calculation

Continuous variables are summarized with the following descriptive statistics: n (number of observations), arithmetic mean (A_mean_), geometric mean (G_mean_), SD, coefficient of variation (CV%), minimum value, median, and maximum value. Categorical data are summarized with frequencies and percentages.

The Sample’s size was calculated assuming an intrasubject coefficient of variation (ISCV) of 40% for AUC_0-t_ of albendazole, which was the highest ISCV observed for the main pharmacokinetic parameters; a true Test-to-Reference GMR of 0.95; a significance level (alpha error) of 5%; and an *a priori* statistical power of 80%, a sample of 66 evaluable subjects was estimated. To compensate for potential dropouts, it was planned to recruit 78 subjects.

Statistical and pharmacokinetic analyses were performed according to EMA’s applicable guidelines, using SAS^®^ 9.4 (SAS Institute Inc., Cary, NC, United States) and Phoenix^®^ WinNonlin^®^ 8.2 (Certara United States Inc., Princeton, NJ), respectively.

## Results

**FIGURE 1:**
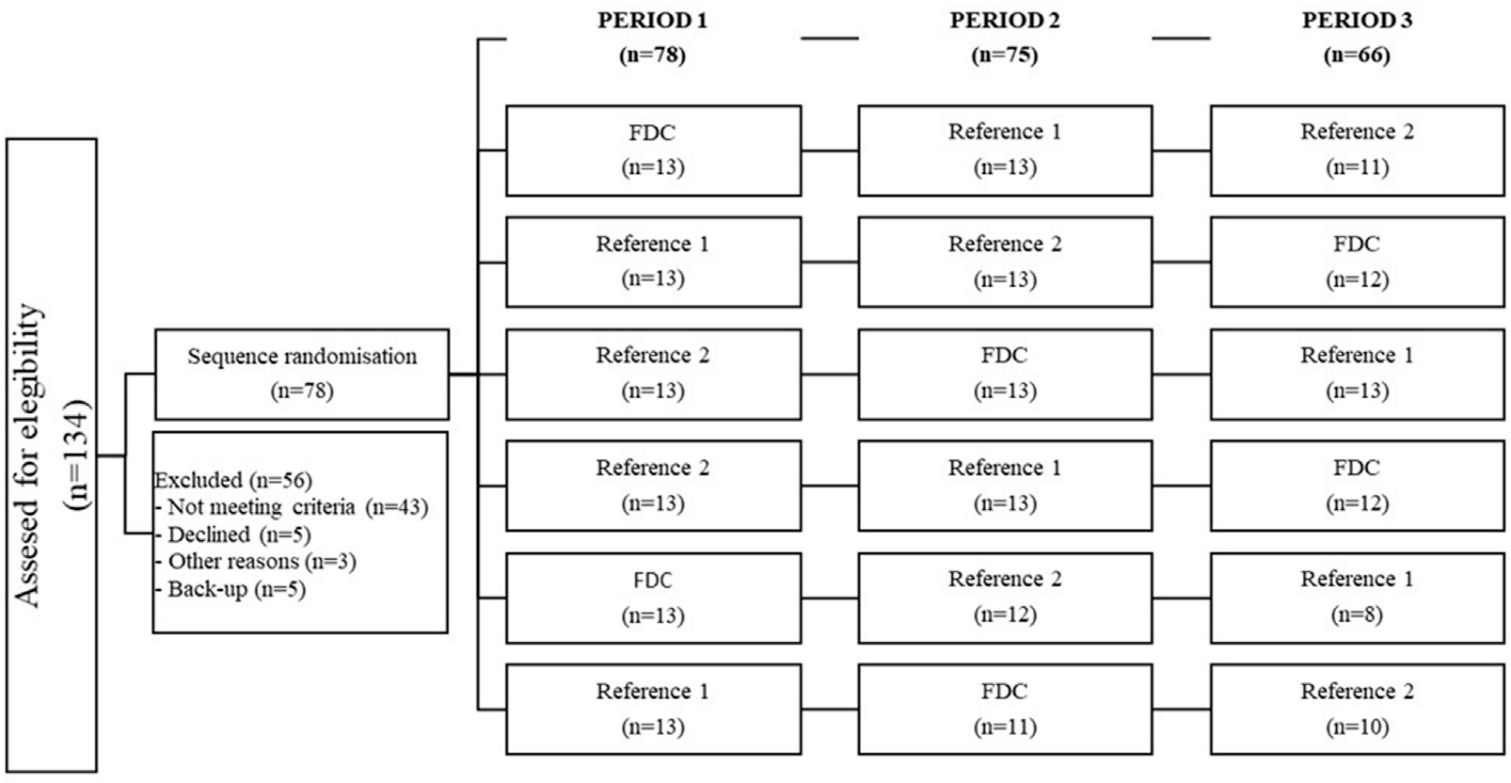
Subject’s allocation.

The subject’s allocation is summarized in Figure 1. A total of 134 subjects was evaluated for eligibility, but 56 were not admitted to randomization due to the following reasons: 43 subjects did not meet one or more of the selection criteria (42 at screening and 1 at admission), 1 subject reported an AE, 5 subjects decided to discontinue after the screening procedures, 2 subjects were discontinued according to the physician’s decision, and 5 subjects served as the back-up. Finally, 78 subjects (35 women and 43 men, aged 19–59 years) were randomized in accordance with a crossover design in one of the six sequences. Seventy-eight subjects completed period 1, 75 subjects completed period 2, and 66 subjects completed the three periods of the study. For comparative bioequivalence (ivermectin and albendazole), 70 subjects were analyzed. Reasons for discontinuation were withdrawal of consent by the subject (4), adverse events (4), failure to meet admission criteria (3), or physician decision (1).

**FIGURE 2:**
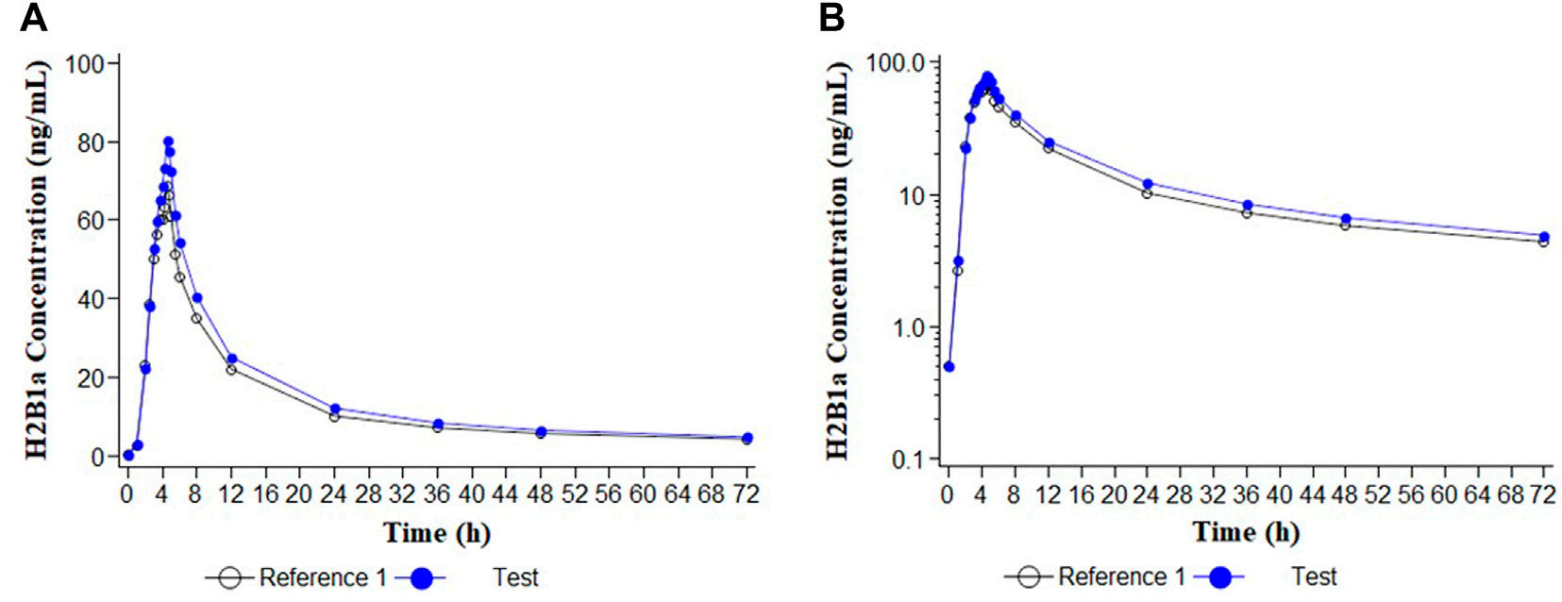
Concentration/time curves for ivermectin H2B1a following the administration of ivermectin/albendazole 18/400 mg tablets (Test) and Ivermectin 6 × 3 mg tablets (Reference 1). **(A)** Linear scale; **(B)** Semi-logarithmic scale.

**FIGURE 3:**
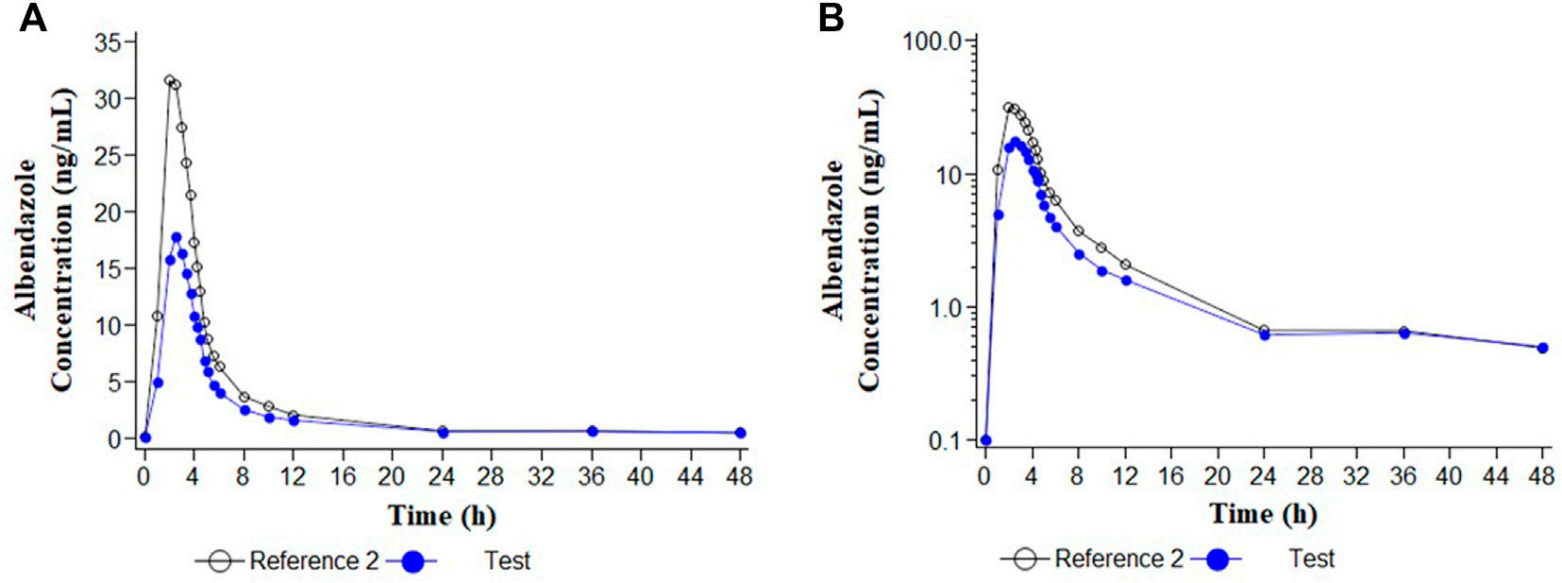
Concentration/time curves for albendazole following the administration of ivermectin/albendazole 18/400 mg tablets (Test) and albendazole tablets (Reference 2). **(A)** Linear scale; **(B)** Semi-logarithmic scale.

Descriptive statistics of the main pharmacokinetic parameters for the four analytes of interest are given in Tables 1, 2. The results of the comparative bioavailability analysis are given in Table 3 and the arithmetic mean of concentration versus time curves are shown in both, linear and semi-logarithmic scales in Figures 2, 3. The bioequivalence was demonstrated for ivermectin since the 90% confidence interval of the two main pharmacokinetic parameters (C_max_ and AUC_0-72_) lay within the 80%–125% acceptance interval. However, bioequivalence was not demonstrated in the case of albendazole since both required pharmacokinetic parameters (C_max_ and AUC_t_) laid below the formal acceptance interval.

**TABLE 1:** Descriptive statistics of the main pharmacokinetic parameters of ivermectin H2B1a and ivermectin H2B1b following the administration of ivermectin/albendazole fixed-dose combination (FDC) or ivermectin (REFERENCE 1).

		FDC	Reference 1
	N	75	70
Ivermectin H2B1a	**C**_**max**_ **(ng/ml)**	86.40 (30.42–139.23)	74.83 (36.67–127.75)
	**AUC**_**0-72**_ **(ng·h/mL)**	1,040 (530–1,678)	905 (506–1,562)
	**t**_**max**_ **(h)**	4.50 (2.50–5.50)	4.50 (2.50–5.50)
	**λ**_**z**_ **(1/h)**	0.018 (0.006–0.038)	0.017 (0.005–0.044)
	**t**_**1/2**_ **(h)**	46.06 (18.34–110.91)	50.50 (15.74–137.44)
	**V/F (L)**	881 (345–2054)	1,046 (463–1808)
	**Cl/F (L/h)**	14.27 (18.15–25.22)	16.46 (7.25–24.81)
	**MRT**_**0-tlast**_ **(h)**	21.20 (18.15–25.22)	20.99 (16.13–24.81)
	**MRT**_**0-∞**_ **(h)**	50.89 (25.27–121.11)	55.55 (20.5–153.01)
Ivermectin H2B1b	**C**_**max**_ **(ng/ml)**	1.49 (0.54–4.06)	0.90 (0.48–1.63)
	**AUC**_**0-t**_ **(ng·h/mL)**	21.96 (9.33–139.12)	12.37 (6.60–21.97)
	**t**_**max**_ **(h)**	4.50 (2.00–72.00)	4.38 (2.00–5.50)
	**λ**_**z**_ **(1/h)**	0.014 (0.003–0.033)	0.015 (0.005–0.050))
	**t**_**1/2**_ **(h)**	64.03 (21.09–222.74)	62.89 (13.80–146.57)
	**V/F (L)**	609 (255–1,063)	1,010 (350–2,402)
	**Cl/F (L/h)**	24.89 (21.10–51.67)	24.11 (14.42–28.52)
	**MRT**_**0-tlast**_ **(h)**	76.39 (31.14–279.69)	78.57 (19.96–187.83)
	**MRT**_**0-∞**_ **(h)**	1.50 (0.54–4.06)	0.90 (0.48–1.63)

Results are expressed as Arithmetic mean (Minimum—Maximum) or Median (Minimum—Maximum) in the case or t_max_.

C_max_, Maximum plasma concentration; AUC_0-72_, Area under the curve from time zero to 72 h; tmax, Time to maximum observed concentration; λz, Apparent terminal elimination rate constant; t_1/2_, Apparent terminal half life; V/F, Apparent volume of distribution after oral administration; Cl/F, Apparent total plasma clearance after oral administration; MRT_0-tlast_, Mean residence time from time zero to last quantifiable time; MRT_0-i_, Mean residence time from time zero extrapolate to infinity.

**TABLE 2:** Descriptive statistics of the main pharmacokinetic parameters of albendazole and albendazole sulfoxide following the administration of ivermectin/albendazole fixed-dose combination (FDC) or albendazole (REFERENCE 2).

		FDC	Reference 2
	N	75	70
Albendazole	**C**_**max**_ **(ng/ml)**	22.27 (1.89–111.78)	37.78 (2.83–141.19)
	**AUC**_**0-72**_ **(ng·h/mL)**	94.65 (11.65–507.78)	147.86 (14.43–529.36)
	**t**_**max**_ **(h)**	2.50 (1.00–12.00)	2.50 (1.00–4.50)
	**λ**_**z**_ **(1/h)**	0.058 (0.012–0.170)	0.066 (0.021–0.184)
	**t**_**1/2**_ **(h)**	17.63 (4.07–59.72)	13.50 (3.76–33.12)
	**V/F (L)**	138,868 (7,087–1,051,291)	81,702 (93,400–469,463)
	**Cl/F (L/h)**	5,416 (782–30,736)	3,846 (741–11,509)
	**MRT**_**0-tlast**_ **(h)**	10.48 (4.11–19.24)	9.11 (3.69–20.04)
	**MRT**_**0-∞**_ **(h)**	17.97 (4.26–76.67)	11.82 (3.77–33.29)
Albendazole sulfoxide	**C**_**max**_ **(ng/ml)**	318.29 (97.12–776.18)	429.62 (84.00–1,103.35)
	**AUC**_**0-t**_ **(ng·h/mL)**	3,916 (974–11,926)	4,883 (1,277–11,572)
	**t**_**max**_ **(h)**	3.33 (2.00–24.00)	3.67 (1.00–4.75)
	**λ**_**z**_ **(1/h)**	0.049 (0.008–0.123)	0.056 (0.018–0.136)
	**t**_**1/2**_ **(h)**	17.91 (5.64–8,390)	14.87 (5.11–39.51)
	**V/F (L)**	2,656 (629–7,562)	2002 (450–10,101)
	**Cl/F (L/h)**	110.10 (39.32–402.69)	88.85 (32.85–224.41)
	**MRT**_**0-tlast**_ **(h)**	14.63 (8.79–21.95)	13.79 (8.21–20.11)
	**MRT**_**0-∞**_ **(h)**	23.25 (9.19–106.08)	19.37 (8.51–36.49)

Results are expressed as Arithmetic mean (Minimum—Maximum) or Median (Minimum—Maximum) in the case or t_max_.

C_max_, Maximum plasma concentration; AUC_0-72_, Area under the curve from time zero to 72 h; tmax, Time to maximum observed concentration; λz, Apparent terminal elimination rate constant; t_1/2_, Apparent terminal half life; V/F, Apparent volume of distribution after oral administration; Cl/F, Apparent total plasma clearance after oral administration; MRT_0-tlast_, Mean residence time from time zero to last quantifiable time; MRT_0-i_, Mean residence time from time zero extrapolate to infinity.

**TABLE 3:** Estimation of the bioequivalence for ivermectin (ivermectin/albendazole FDC vs. ivermectin) and albendazole (ivermectin/albendazole FDC vs. albendazole).

	Parameter	FDC (Geom.mean)	Reference (Geom.mean)	T/R ratio	90% confidence interval	Outcome
Ivermectin	**C**_**max**_	83.5	72.31	118.48	110.68–120.49	Bioequivalent
	**AUC**_**0-72**_	1,000.09	870.12	114.94	110.46–119.60	Bioequivalent
Albendazole	**C**_**max**_	15.74	25.76	61.11	53.10–70.34	No bioequivalent
	**AUC**_**0-t**_	74.42	108.80	68.40	61.13–76.54	No bioequivalent

C_max_, Maximum plasma concentration; AUC_0-72_, Area under the curve from time zero to 72 h; AUC_0-t_, Aurea under the curve from time zero to 48 h post-administration.

No deaths or severe adverse events were reported. Thirty-seven (37) out of the 78 subjects who received at least one dose of the investigational medication reported a total of 62 adverse events, being considered as mild (51) or moderate (11) in intensity. No difference was observed in the number of subjects reporting adverse events between treatments (ivermecin/albendazole FDC, 17%; ivermectin, 29%; albendazole, 18%). In all three groups, the most common event was headache (16), followed by abdominal pain (2), diarrhea, nausea (2), and somnolence (2). Four subjects withdrew from the follow-up due to hyperthermia, nausea and myalgia, allergic reaction, and urinary tract infection, all of them were considered as not drug-related. No relevant abnormalities in the clinical laboratory measurements, vital signs, electrocardiograms, or physical examinations were observed.

## Discussion

The final objective of the STOP consortium is to provide target countries with a new medicine to treat a neglected disease. Then, the regulatory pathway selected was the EMA procedure to review medicines for use outside the European Union (now called “M4all,” formerly named “Article 58”). Through this procedure, the EMA, in cooperation with the World Health Organization, can provide a scientific opinion on high-priority human medicines intended to be used outside the European Union, with the aim to facilitate patient access to essential medicines in low- and middle-income countries, including new or improved therapies for unmet medical needs, which are intended to prevent or treat diseases of major public health interest. The benefit of this procedure is to obtain a rigorous scientific assessment by EMA, WHO, and other experts of the same high standards as for medicines intended for use in Europe, to facilitate the registration in target countries (Cavaller Bellaubi et al., 2020). The EMA accepted to review this product in 2018, and since then, several scientific advices were conducted to agree on the clinical plan and to discuss the design of the contained studies (including this one).

As a consequence, this is the first clinical trial of the clinical development of an innovative fixed-dose combination of ivermectin/albendazole 18/400 and 9/400 mg. As described by the EMA guidance (European Medicines Agency, 2010) for products with several strengths that follow a linear pharmacokinetic, only the bioavailability of the highest strength (18/400 mg) was assessed. The trial was conducted with the objective of characterizing the pharmacokinetic profile of this new formulation and to compare with the corresponding references already marketed, in accordance with the EMA guidelines on the clinical development of fixed-dose combination medicinal products (European Medicines Agency, 2017), which requires the demonstration of similar pharmacokinetics (usually through demonstrating bioequivalence) of the fixed-dose combination medicinal product versus its individual active substances taken simultaneously. The subsequent clinical program has been recently published (Krolewiecki et al., 2022) and will include a phase II trial to reveal the safety of the investigational product in children above 15 kg body weight. In case of a positive outcome, a sub-sequent phase III clinical trial to demonstrate the efficacy and safety will be carried out. In addition, during the clinical development, further outcomes, such as the palatability and acceptability of the new drug or its contribution to reduce the anti-helminthic resistance to pharmacotherapy, will be also evaluated.

The investigation of drug–drug interactions is usually required within the development of a fixed combination (European Medicines Agency, 2017), however, it was not necessary in this case because it was already investigated. Awadzi et al. (2003) studied the pharmacokinetic interaction between ivermectin and albendazole sulphoxide in patients with onchocerciasis, and concluded that no significant interaction was shown. The lack of interaction was later confirmed by other studies (Na-Bangchang et al., 2006; González Canga et al., 2008). The selection of the dose of 400 mg of albendazole is fully justified because this is the recommended fixed-dose regimen for preventive chemotherapy as public health intervention in subjects over 12 months of age. In the case of ivermectin, the recommended dosage should be adjusted for body weight, usually at 0.2–0.4 mg/kg, then requiring the individualization of the dose. In the scenario of mass drug administration programs, the need to weight each patient and then calculate the dosage, was considered “labor- and time-intensive,” and hence, a constraint (Thylefors et al., 2008). Due to the higher complexity of dose adjustment, fixed-dose regimens are preferred for facilitating large-scale treatment programs and community-based treatments by non-medical personnel (Gabrielli et al., 2011). Then, the intended dosage in the present project ranges up to 600 mcg/kg in a fixed-dose regimen, because this dosage has demonstrated its safety in various indications (Navarro et al., 2020), even in small children (Smit et al., 2018; Matamoros et al., 2021). Nevertheless, as mentioned previously, a therapeutic exploratory (phase II), dose-escalating clinical trial will be conducted to clearly establish the safety of this dosage with the new formulation in small children prior to entering a large therapeutic confirmatory (phase III) trial.

Another advantage of a fixed-dose combination is certain since it can offer a more effective therapeutic effect due to the synergistic and/or additive effect, mainly in the case of patients affected by several parasites. And, from a practical perspective, when compared to the co-administration of drugs, the fixed-dose combination is more convenient, mainly considering the case of mass drug administration. Due to these advantages, fixed-dose combinations are routinely used in developing countries for the treatment of infectious diseases such as HIV or *tuberculosis* and are recommended as an alternative therapy for neglected tropical diseases (Ferraz et al., 2022). In the event of STH, it is well established that the poor efficacy of benzimidazoles against *T. trichiura* and *S. stercolaris* is significantly improved by the co-administration of ivermectin (Matamoros et al., 2021; Knopp et al., 2010).

In the particular case of this new ivermectin/albendazole innovative combination, it has the additional advantage of being orodispersible. A recent document from WHO addresses the topic of the safe administration of medicines for treatment of neglected tropical diseases, with a focus on mass drug administration (MDA) (World Health Organization, 2021c). This document raises concerns about the deaths from choking, primarily in young children, which are related to how medicines are administered rather than to their pharmacology, suggesting that forcing children to swallow tablets against their will is the main risk factor for choking. One of the more adequate ways to avoid this problem is the use of an orodispersible formulation as the FDC hereby proposed.

The present study was conducted to characterize the pharmacokinetic profile of the new FDC, in comparison with each of the drugs separately. Following the recommendation by the EMA guidelines on bioequivalence (European Medicines Agency, 2010), the highest strength was assayed (ALB/IVM 400 mg/18 mg).

The study was conducted in adult healthy volunteers, which is the more appropriate population to describe the pharmacokinetic parameters at this stage of clinical development. Noteworthily, to complete this information, an additional population pharmacokinetic study in infected children is included in the protocol of the next clinical trial (phase II) of the clinical development.

For ivermectin, the SmPC of the reference product requires the administration of the product with water, in an empty stomach (no food should be taken within 2 hours before or after administration). For albendazole, the SmPC of the reference product requires the administration of the product on a fed state. The FDC is intended to be used mainly under mass drug administration programs, where the control of the fast or fed state is difficult and always a challenge in developing countries. Therefore, the pharmacokinetic trial hereby described was conducted after a light meal, as representative of the usual condition on the field.

There were no protocol amendments and no relevant changes in the study conduct and analyses described in the protocol. The study design was demonstrated as appropriate, i.e., the washout period was enough to prevent any carryover effect and the selected sampling times were appropriated for the characterization of both, the absorption and elimination phases of the plasma concentration/time curve, and therefore, to allow the estimation of pharmacokinetic parameters. In general, ivermectin’s (Smit et al., 2018) and albendazole’s (Ceballos et al., 2018) pharmacokinetic profiles were similar to other studies that used a similar dosage.

Regarding the pharmacokinetic characterization of the fixed-dose combination, the pharmacokinetic profile of the co-administration of ivermectin and albendazole was investigated by three different studies. As stated previously, Awadzi et al. (1994) studied the pharmacokinetic interaction of ivermectin and albendazole sulphoxide, alone and in co-administration, but unfortunately the pharmacokinetic parameters were not disclosed as to allow a comparison with our results. Na-Bangchang et al. (2006) evaluated the pharmacokinetic drug interactions of ivermectin, albendazole, and praziquantel in healthy Thai volunteers. In one of the arms, ivermectin (0.2 mg/kg) was given concurrently with albendazole (400 mg), under a fasting condition. In the case of albendazole sulphoxide, the main parameters showing bioavailability (Cmax, AUC) were slightly lower than in this study, probably due to the improved absorption of albendazole when given with a meal (Awadzi et al., 1994). In the case of ivermectin, a slightly higher bioavailability was also shown in our study, attributable to the higher dosage administered. Finally, Thomsen et al. (2016) compared the pharmacokinetics of co-administered ivermectin, albendazole, and diethylcarbamazine in bancroftian filariasis. Unfortunately, pharmacokinetic data were not comparable with the obtained data in our study because of the concurrent administration of the third drug (diethylcarbamazine).

The results of the comparative bioavailability have demonstrated the bioequivalence of the test product ivermectin/albendazole FDC versus the reference ivermectin. However, the bioequivalence could not be demonstrated for albendazole, since the 90% for the ratio of the main pharmacokinetic parameters’ confidence interval lay out of the formal acceptance range of 80.00%–125.00%. The reduced bioavailability in the case of FDC could potentially be due to the fact that albendazole tablets (reference formulation) were swallowed with water whereas FDC was given without water in accordance with the appropriate guidelines (European Medicines Agency, 2010), and also as expected for an orodispersible formulation intended to be taken “in real life” without water (worthy in situations where access to drinking water is especially difficult).

Nonetheless, the lack of demonstration of formal bioequivalence in the case of albendazole will not preclude progressing to the next phase of clinical development (a phase II, dose-finding trial) where the pharmacokinetic profile of the FDC will continue being characterized through a population pharmacokinetic sub-study in pediatric patients. In addition, it is important to remark that albendazole is poorly absorbed (<5%) and its main anti-helminthic activity is developed intra-intestinally, i.e., albendazole acts directly on luminal parasites in the gastrointestinal tract (Chai et al., 2021).

No relevant safety concerns were raised during the study, and the discontinuations were considered as not related with the treatment, then, the study medication can be deemed as safe and with good tolerability. This finding is in agreement with the well-known safety profile of both, ivermectin and albendazole, either administered separately or co-administered (Knopp et al., 2010; Matamoros et al., 2021; Smit et al., 2018). The fact that, the most common AE reported by far was headaches is not surprising, since it is well-known that headache is the most common adverse event in human pharmacology studies with the confinement of healthy volunteers, regardless the drug (or placebo) received (Sibille et al., 1998). In a large surveillance of safety in healthy participants in phase I research, it was reported that up to 80% of participants receiving placebo experienced a mild adverse event (headache being the most frequent) and it was attributed to changes in the behavior required for the participation in the study, such as abstinence from smoking or drinking alcohol or caffeinated beverages rather than from the study drug (Emanuel et al., 2015). In conclusion, this article describes the first phase of the clinical investigation of a new fixed-dose combination of ivermectin and albendazole for the treatment of helminthiasis, developed to facilitate its usage under mass drug administration programs. The pharmacokinetic profile of the fixed-dose combination was appropriately characterized and compared with each of the individual components. Moreover, the bioequivalence versus the reference ivermectin was demonstrated, though the bioequivalence versus the albendazole reference was not shown. All the three medications analyzed were well tolerated. These results allow the development of the following phase of the clinical development to demonstrate the efficacy and safety in patients affected by soil-transmitted helminths.

## Data Availability

The raw data supporting the conclusion of this article will be made available by the authors, without undue reservation.

## References

[B1] Anderson R. M. Turner H. C. Truscott J. E. Hollingsworth T. D. Brooker S. J. (2015). Should the Goal for the Treatment of Soil Transmitted Helminth (STH) Infections Be Changed from Morbidity Control in Children to Community-wide Transmission Elimination? PLoS Negl. Trop. Dis. 9 (8), e0003897. 10.1371/journal.pntd.0003897 26291538 PMC4546270

[B2] Awadzi K. Edwards G. Duke B. O. Opoku N. O. Attah S. K. Addy E. T. (2003). The Co-administration of Ivermectin and Albendazole-Ssafety, Pharmacokinetics and Efficacy against Onchocerca Volvulus. Ann. Trop. Med. Parasitol. 97 (2), 165–178. 10.1179/000349803235001697 12803872

[B3] Awadzi K. Hero M. Opoku N. O. Büttner D. W. Coventry P. A. Prime M. A. (1994). The Chemotherapy of Onchocerciasis XVII. A Clinical Evaluation of Albendazole in Patients with Onchocerciasis; Effects of Food and Pretreatment with Ivermectin on Drug Response and Pharmacokinetics. Trop. Med. Parasitol. 45, 203–208. 7899788

[B4] Cavaller Bellaubi M. Harvey Allchurch M. Lagalice C. Saint-Raymond A. (2020). The European Medicines Agency Facilitates Access to Medicines in Low- and Middle-Income Countries. Expert Rev. Clin. Pharmacol. 13 (3), 321–325. PMID: 32053756. 10.1080/17512433.2020.1724782 32053756

[B5] Ceballos L. Krolewiecki A. Juárez M. Moreno L. Schaer F. Alvarez L. I. (2018). Assessment of Serum Pharmacokinetics and Urinary Excretion of Albendazole and its Metabolites in Human Volunteers. PLoS Negl. Trop. Dis. 12 (1), e0005945. 10.1371/journal.pntd.0005945 29346367 PMC5773000

[B6] Chai J. Y. Jung B. K. Hong S. J. (2021). Albendazole and Mebendazole as Anti-parasitic and Anti-Cancer Agents: an Update. Korean J. Parasitol. 59 (3), 189–225. 10.3347/kjp.2021.59.3.189 34218593 PMC8255490

[B7] Echazú A. Juarez M. Vargas P. A. Cajal S. P. Cimino R. O. Heredia V. (2017). Albendazole and Ivermectin for the Control of Soil-Transmitted Helminths in an Area with High Prevalence of Strongyloides Stercoralis and Hookworm in Northwestern Argentina: A Community-Based Pragmatic Study. PLoS Negl. Trop. Dis. 11 (10), e0006003. 10.1371/journal.pntd.0006003 28991899 PMC5648268

[B8] Emanuel E. J. Bedarida G. Macci K. Gabler N. B. Rid A. Wendler D. (2015). Quantifying the Risks of Non-oncology Phase I Research in Healthy Volunteers: Meta-Analysis of Phase I Studies. BMJ 350, h3271. 10.1136/bmj.h3271 26115663 PMC4482145

[B9] European Medicines Agency (2017). Guideline on Clinical Development of Fixed Combination Medicinal Products. Available at: https://www.ema.europa.eu/en/documents/scientific-guideline/guideline-clinical-development-fixed-combination-medicinal-products-revision-2_en.pdf (Accessed March 31, 2022).

[B10] European Medicines Agency (2010). Guideline on the Investigation of Bioequivalence. Ref.: CPMP/EWP/QWP/1401/98 Rev.1/Corr^∗∗^ . Available at: https://www.ema.europa.eu/en/documents/scientific-guideline/guideline-investigation-bioequivalence-rev1_en.pdf (Accessed March 31, 2022).

[B11] Ferraz L. R. M. Silva L. C. P. B. B. Souza M. L. Alves L. P. Sales V. A. W. Barbosa I. D. N. G. (2022). Drug Associations as Alternative and Complementary Therapy for Neglected Tropical Diseases. Acta Trop. 225, 106210. 10.1016/j.actatropica.2021.106210 34687644

[B12] Gabrielli A. F. Montresor A. Chitsulo L. Engels D. Savioli L. (20112011). Preventive Chemotherapy in Human Helminthiasis: Theoretical and Operational Aspects. Trans. R. Soc. Trop. Med. Hyg. 105, 683–693. 10.1016/j.trstmh.2011.08.013 PMC5576527 22040463

[B34] González Canga A. Sahagún Prieto A. M. Diez Liébana M. J. Fernández Martínez N. Sierra Vega M. García Vieitez J. J. (2008). The Pharmacokinetics and Interactions of Ivermectin in Humans--a Mini-Review. AAPS J. 10 (1), 42–46. 10.1208/s12248-007-9000-9 18446504 PMC2751445

[B13] Jourdan P. M. Lamberton P. H. L. Fenwick A. Addiss D. G. (2017). Soil-transmitted Helminth Infections. Lancet 6736 (17), 1–14. 10.1016/S0140-6736(17)31930-X 28882382

[B14] Knopp S. Mohammed K. A. Speich B. Hattendorf J. Khamis I. S. Khamis A. N. (20102010). Albendazole and Mebendazole Administered Alone or in Combination with Ivermectin against Trichuris Trichiura: A Randomized Controlled Trial. Clin. Infect. Dis. 51 (12), 1420–1428. 10.1086/657310 21062129

[B15] Krolewiecki A. Enbiale W. Gandasegui J. van Lieshout L. Kepha S. Messa Junior A. (2022). An Adaptive Phase II/III Safety and Efficacy Randomized Controlled Trial of Single Day or Three-Day Fixed-Dose Albendazole-Ivermectin Co-formulation versus Albendazole for the Treatment of Trichuris Trichiura and Other STH Infections. ALIVE Trial Protocol. Gates Open Res. 6, 62. 10.12688/gatesopenres.13615.1 PMC9714317 36540062

[B16] Krolewiecki A. J. Lammie P. Jacobson J. Gabrielli A. F. Levecke B. Socias E. (2013). A Public Health Response against Strongyloides Stercoralis: Time to Look at Soil-Transmitted Helminthiasis in Full. PLoS Negl. Trop. Dis. 7 (5), e2165. 10.1371/journal.pntd.0002165 23675541 PMC3649958

[B17] Lo N. C. Addiss D. G. Hotez P. H. King C. H. Stothard J. R. Evans D. S. (2016). A Call to Strengthen the Global Strategy against Schistosomiasis and Soil-Transmitted Helminthiasis: the Time Is Now. Lancet Infect. Dis. 3099 (16), e64–e69. 10.1016/S1473-3099(16)30535-7 PMC5280090 27914852

[B18] Matamoros G. Sánchez A. Gabrie J. A. Juárez M. Ceballos L. Escalada A. (2021). Efficacy and Safety of Albendazole and High-Dose Ivermectin Coadministration in School-Aged Children Infected with Trichuris Trichiura in Honduras: A Randomized Controlled Trial. Clin. Infect. Dis. 73 (7), 1203–1210. 10.1093/cid/ciab365 33906234

[B19] Moser W. Schindler C. Keiser J. (2017). Efficacy of Recommended Drugs against Soil Transmitted Helminths: Systematic Review and Network Meta-Analysis. BMJ 358, j4307. 10.1136/bmj.j4307 28947636 PMC5611648

[B20] Na-Bangchang K. Kietinun S. Pawa K. K. Hanpitakpong W. Na-Bangchang C. Lazdins J. (2006). Assessments of Pharmacokinetic Drug Interactions and Tolerability of Albendazole, Praziquantel and Ivermectin Combinations. Trans. R. Soc. Trop. Med. Hyg. 100 (4), 335–345. 10.1016/j.trstmh.2005.05.017 16271272

[B21] Navarro M. Camprubí D. Requena-Méndez A. Buonfrate D. Giorli G. Kamgno J. (2020). Safety of High-Dose Ivermectin: a Systematic Review and Meta-Analysis. J. Antimicrob. Chemother. 75 (4), 827–834. 10.1093/jac/dkz524 31960060

[B22] Richards F. O. (2017). Upon Entering an Age of Global Ivermectin-Based Integrated Mass Drug Administration for Neglected Tropical Diseases and Malaria. Malar. J. 16 (1), 168. 10.1186/s12936-017-1830-z 28438168 PMC5404338

[B23] Sibille M. Deigat N. Janin A. Kirkesseli S. Durand D. V. (1998). Adverse Events in Phase-I Studies: a Report in 1015 Healthy Volunteers. Eur. J. Clin. Pharmacol. 54 (1), 13–20. 10.1007/s002280050413 9591924

[B24] Smit M. R. Ochomo E. O. Aljayyoussi G. Kwambai T. K. Abong'o B. O. Chen T. (2018). Safety and Mosquitocidal Efficacy of High-Dose Ivermectin when Co-administered with Dihydroartemisinin-Piperaquine in Kenyan Adults with Uncomplicated Malaria (IVERMAL): a Randomised, Double-Blind, Placebo-Controlled Trial. Lancet Infect. Dis. 18 (6), 615–626. 10.1016/S1473-3099(18)30163-4 29602751

[B25] The 2017 Global Burden DiseaseInjury IncidencePrevalence Collaborators (2018). Global, Regional, and National Incidence, Prevalence, and Years Lived with Disability for 354 Diseases and Injuries for 195 Countries and Territories, 1990-2017: a Systematic Analysis for the Global Burden of Disease Study 2017. Lancet 392 (10159), 1789–1858. 10.1016/S0140-6736(18)32279-7 30496104 PMC6227754

[B26] Thomsen E. K. Sanuku N. Baea M. Satofan S. Maki E. Lombore B. (2016). Efficacy, Safety, and Pharmacokinetics of Coadministered Diethylcarbamazine, Albendazole, and Ivermectin for Treatment of Bancroftian Filariasis. Clin. Infect. Dis. 62 (3), 334–341. 10.1093/cid/civ882 26486704

[B27] Thylefors B. Alleman M. M. Twum-Danso N. A. (2008). Operational Lessons from 20 Years of the Mectizan Donation Program for the Control of Onchocerciasis. Trop. Med. Int. Health 13 (5), 689–696. 10.1111/j.1365-3156.2008.02049.x 18419585

[B28] Vercruysse J. Albonico M. Behnke J. M. Kotze A. C. Prichard R. K. McCarthy J. S. (2011). Is Anthelmintic Resistance a Concern for the Control of Human Soil-Transmitted Helminths? Int. J. Parasitol. Drugs. Drug. Resist. 1 (1), 14–27. 10.1016/j.ijpddr.2011.09.002 24533260 PMC3913213

[B29] World Health Organization (2021b). Notes on the Design of Bioequivalence Study: Albendazole. Available at: https://extranet.who.int/pqweb/sites/default/files/documents/BE_Albendazole_March2021.pdf (Accessed March 31, 2022).

[B30] World Health Organization (2021a). Notes on the Design of Bioequivalence Study: Ivermectin. Available at: https://extranet.who.int/pqweb/sites/default/files/documents/BE_ivermectin_July2021.pdf (Accessed March 31, 2022).

[B31] World Health Organization (2021c). Safety in Administering Medicines for Neglected Tropical Diseases. Available at: https://www.who.int/publications/i/item/9789240024144 (Accessed March 31, 2022).

[B32] World Health Organization (2022a). WHO Model List of Essential Medicines (EML). Available at: https://list.essentialmeds.org/?query=ivermectin (Accessed March 31, 2022).

[B33] World Health Organization (2022b). WHO Model List of Essential Medicines (EML). Available at: https://list.essentialmeds.org/?query=albendazole (Accessed March 31, 2022).

